# Comparative analysis of background EEG activity in juvenile myoclonic epilepsy during valproic acid treatment: a standardized, low-resolution, brain electromagnetic tomography (sLORETA) study

**DOI:** 10.1186/s12883-022-02577-6

**Published:** 2022-02-09

**Authors:** Ja-Un Moon, Joo-Young Lee, Kwang-Yeon Kim, Tae-Hoon Eom, Young-Hoon Kim, In-Goo Lee

**Affiliations:** Department of Pediatrics, College of Medicine, The Catholic University of Korea, Seoul, Republic of Korea

**Keywords:** Juvenile myoclonic epilepsy (JME), Quantitative electroencephalography (qEEG), Distributed source model, Valproic acid, Standardized low-resolution brain electromagnetic tomography (sLORETA)

## Abstract

**Background:**

By definition, the background EEG is normal in juvenile myoclonic epilepsy (JME) patients and not accompanied by other developmental and cognitive problems. However, some recent studies using quantitative EEG (qEEG) reported abnormal changes in the background activity. QEEG investigation in patients undergoing anticonvulsant treatment might be a useful approach to explore the electrophysiology and anticonvulsant effects in JME.

**Methods:**

We investigated background EEG activity changes in patients undergoing valproic acid (VPA) treatment using qEEG analysis in a distributed source model. In 17 children with JME, non-parametric statistical analysis using standardized low-resolution brain electromagnetic tomography was performed to compare the current density distribution of four frequency bands (delta, theta, alpha, and beta) between untreated and treated conditions.

**Results:**

VPA reduced background EEG activity in the low-frequency (delta-theta) bands across the frontal, parieto-occipital, and limbic lobes (threshold log-*F*-ratio = ±1.414, *p* < 0.05; threshold log-*F*-ratio= ±1.465, *p* < 0.01). In the delta band, comparative analysis revealed significant current density differences in the occipital, parietal, and limbic lobes. In the theta band, the analysis revealed significant differences in the frontal, occipital, and limbic lobes. The maximal difference was found in the delta band in the cuneus of the left occipital lobe (log-*F*-ratio = −1.840) and the theta band in the medial frontal gyrus of the left frontal lobe (log-*F*-ratio = −1.610).

**Conclusions:**

This study demonstrated the anticonvulsant effects on the neural networks involved in JME. In addition, these findings suggested the focal features and the possibility of functional deficits in patients with JME.

## Background

Juvenile myoclonic epilepsy (JME) is the most common and well-defined generalized epilepsy syndrome. It usually begins in adolescence, with a peak onset between 12 and 18 years [1, 2]. JME is characterized by myoclonic seizures, especially shortly after awakening. Generalized tonic-clonic seizures often occur, and one-third of patients also have absence seizures [3]. Interictal and ictal encephalography (EEG) typically show rapid (4-6Hz) spike-waves and polyspike-waves, and photosensitivity occurs in 30% of the patients [3, 4]. Valproic acid (VPA) is the treatment drug of choice and effectively controlled all seizures in about 80 – 90% of the patients [3, 4].

By definition, the background EEG is normal in JME patients and not accompanied by other developmental and cognitive problems [1, 4]. However, some recent studies using quantitative EEG (qEEG) analysis reported abnormal changes in background activity in JME patients [5–7]. In addition, neuropsychological studies showed cognitive dysfunction [8–10], and several studies using multimodal methods such as magnetoencephalography (MEG), functional magnetic resonance imaging (fMRI), and evoked potentials have supported these abnormalities in JME [11–15].

QEEG analysis is an excellent tool to evaluate background EEG and is more sophisticated, with brain-mapping techniques [5, 16, 17]. In brain mapping, the distributed source model has underlying advantages, and its algorithms address the inverse problem with few lead-in assumptions [18]. However, only a few studies have investigated the EEG characteristics of JME using a distributed model [7, 19]. Moreover, background EEG changes in patients undergoing antiepileptic drug (AED) treatment have rarely been quantified. QEEG investigation in patients undergoing AED treatment might be a useful approach to explore the electrophysiological characteristics and AED effects in JME. To better understand neurophysiological alterations and their progress in JME, the present study investigated changes in background EEG activity during treatment with VPA, the drug of choice in JME. We performed a comparative qEEG analysis of background EEG activity by a distributed source model in patients undergoing VPA treatment.

## Methods

### Patients and EEG recordings

A total of 17 patients (7 males, 10 females), newly diagnosed with JME and not receiving anticonvulsant treatment from January 2018 to December 2020 were enrolled in this study. The mean age at diagnosis was 13.9 ± 2.1 years (standard deviation; SD), ranging from 7 to 16 years. The diagnosis of JME was based on the International League Against Epilepsy (ILAE) classification [20]. The details of the EEG recording were the same as those used in previous our study [21–23]. The first EEG was recorded in a drug-free state. The initial daily VPA dose was 15 mg/kg and progressively increased until the seizure was controlled. A second EEG was recorded at 6 months after the seizure was controlled. At the time of the second EEG, the daily dose of VPA was 22.3 ± 4.4 mg/kg (mean±SD). All participants and their parents provided written informed consent prior to the data acquisition [21–23].

EEG recordings were carried out for 30 minutes using a NicoletOne^TM^ EEG system (Natus Medical Inc.; Pleasanton, CA, USA) with a sampling rate of 500 Hz. The EEG recordings were performed according to guidelines of the American Clinical Neurophysiology Society (ACNS) [24]. Activation procedures were performed with intermittent photostimulation (at 5, 10, 15, 20, and 25 Hz) and hyperventilation (3 minutes). Twenty-one Ag/AgCl electrodes were placed according to the international 10-20 system, including the standard 16 temporal and parasagittal scalp sites along with Fz, Cz, Pz, A1, and A2. Additional electrodes for artifact identification were also employed, including two sites near the eyes, plus recordings of respiration and electrocardiography. Nineteen-channel EEG was recorded using a linked-ears reference. Additional bipolar montages were used to differentiate between EEG and eye movement potentials and detect electromyographic activity. Electrode impedance did not exceed 5 kΩ. In the EEG derivations, the filters were set at 1.0 and 70 Hz. Sixteen-bit online digitization was used [21–23].

### EEG data processing and analysis

Principle of EEG data processing and analysis based on our previous study [21–23]. A comparative analysis of background EEG activity was performed using the fast Fourier transform (FFT) technique on 3-s manually segmented, artifact-free epochs (at rest without non-stationary elements, such as epileptiform or paroxysmal discharges) in EEG recordings after a visual inspection. This epoch length was adequate to compute an FFT and short enough to include a sufficient number of artifact-free segments. For each patient, a dataset of 20 epochs was collected for each examination. The segments were representative as they were (1) chosen randomly across the whole length of the EEG recording and (2) representative of the whole recording. The epochs were selected blindly by one author and independently reviewed by a second author. The epochs were collected for four frequency bands (delta, 1.5 – 3.5 Hz; theta, 3.5 –7.5 Hz; alpha, 7.5 – 12.5 Hz; and beta, 12.5 – 25.0 Hz). The limits of each frequency band were referenced from relevant prior studies [21–23, 25, 26]. The EEG recordings were exported into American Standard Code for Information Interchange (ASCII) files and imported into standardized low-resolution brain electromagnetic tomography (sLORETA) software.

The current density distributions in the untreated and treated conditions were compared by voxel-by-voxel analysis of the sLORETA data for four frequency bands. Statistical non-parametric mapping (SnPM) of sLORETA data was used to compare the untreated and treated conditions for four frequency bands [16, 27, 28]. SnPM of sLORETA was performed multiple times with all electrodes or voxels, and for all time samples and discrete frequencies. SnPM of the sLORETA images was performed for each contrast with the built-in voxel-wise randomization tests (5000 permutations in the present study) and employed a log-*F*-ratio statistic for dependent groups with thresholds of *p* < 0.05 and < 0.01, corrected for multiple comparisons. The correction for multiple comparisons in SnPM with random permutations has been shown to yield results similar to those obtained from statistical parametric mapping with a general linear model with multiple comparison corrections derived from random field theory [27, 28].

In the sLORETA images, the cortex is modeled as a collection of volume elements (6239 voxels, size 5 × 5 × 5 mm), restricted to the cortical grey matter, hippocampus, and amygdala in the digitized Montreal Neurological Institute (MNI) coordinates corrected to the Talairach coordinates [16, 29]. Scalp electrode coordinates on the MNI brain were derived from the international 5% system [30].

## Results

VPA decreased background EEG activity in the delta and theta frequency bands (threshold log-*F*-ratio = ±1.414, *p* < 0.05; threshold log-*F*-ratio= ±1.465, *p* < 0.01).

**Fig. 1:**
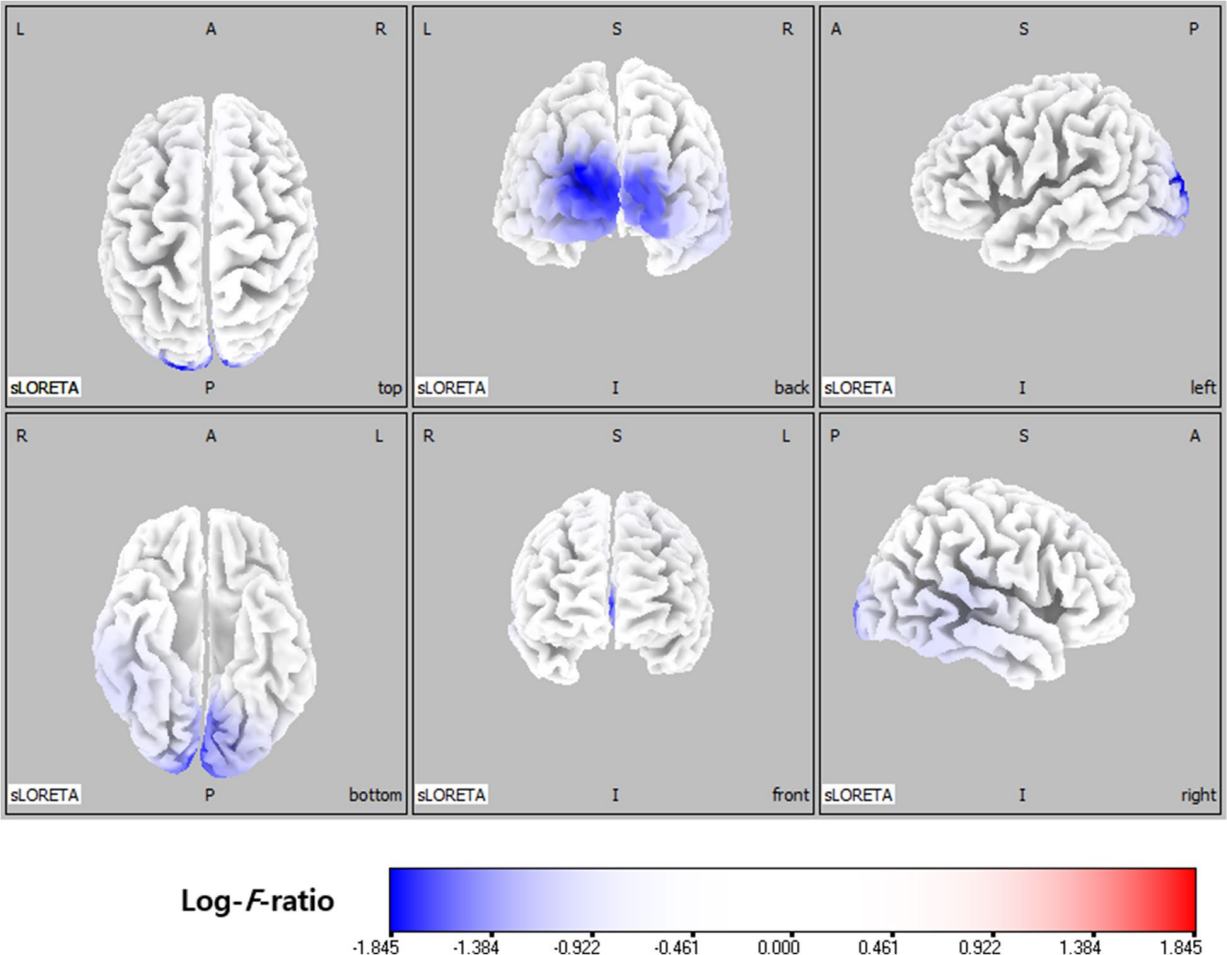
Statistical maps using standardized low-resolution brain electromagnetic tomography (sLORETA) of the delta frequency band during valproic acid treatment were projected onto a three-dimensional fiducial brain cortex. Non-parametric statistical analysis was performed to compare the current density distributions between the untreated and treated conditions. The colored areas represent the spatial extent of the voxels with a significant difference in current density. Log-*F*-ratio statistics were employed, and the color scale represents log-*F*-ratio values (threshold log-*F*-ratio = ±1.414, *p* < 0.05; threshold log-*F*-ratio= ±1.465, *p* < 0.01). A indicates anterior; *P* posterior, *S* superior, *I* inferior, *L* left, *R* right

**Fig. 2:**
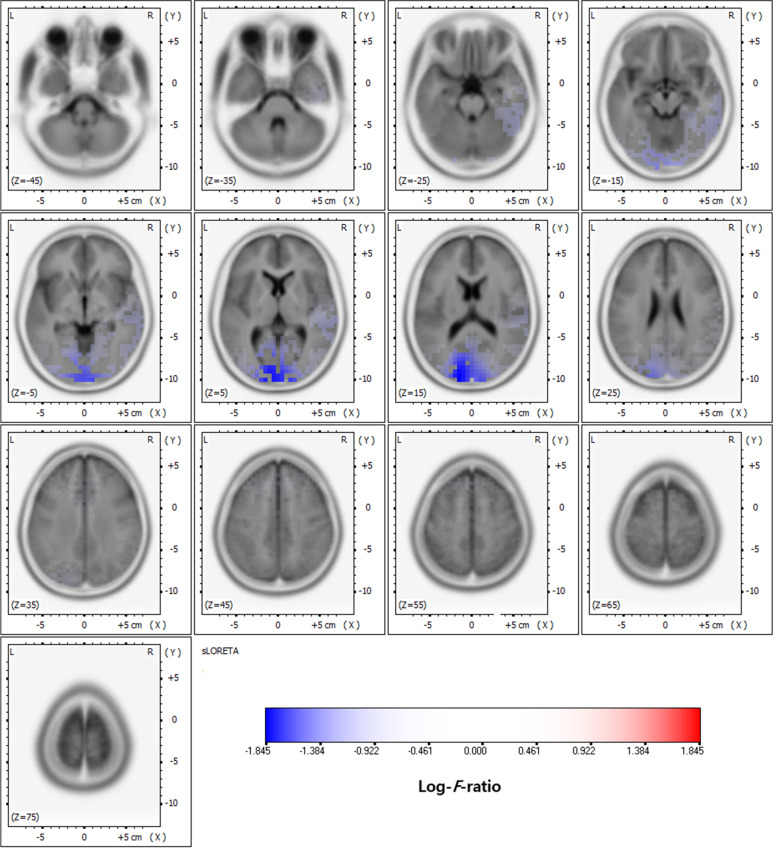
Statistical maps using standardized low-resolution brain electromagnetic tomography (sLORETA) of the delta frequency bands during valproic acid treatment were projected onto a brain magnetic resonance imaging (MRI) template. Nonparametric statistical analysis was performed to compare the current density distributions between the untreated and treated conditions. . The colored areas represent the spatial extent of the voxels with a significant difference in current density. Log-F-ratio statistics were employed, and the color scale represents log-F-ratio values (threshold log-F-ratio = ±1.414, *p* < 0.05; threshold log-F-ratio= ±1.465, *p* < 0.01). L indicates left; R right

**Fig. 3:**
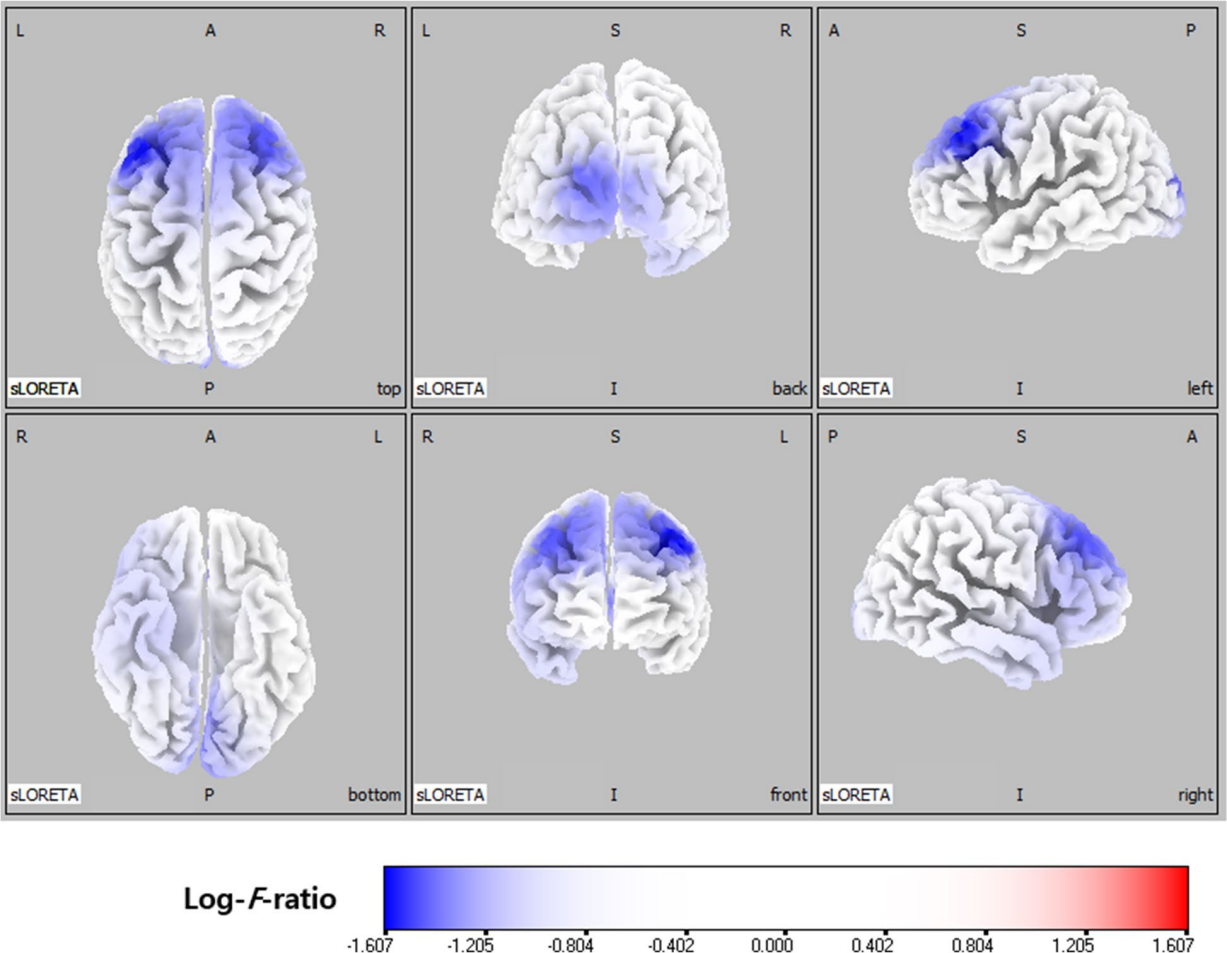
Statistical maps using standardized low-resolution brain electromagnetic tomography (sLORETA) of the theta frequency band during valproic acid treatment were projected onto a three-dimensional fiducial brain cortex. Non-parametric statistical analysis was performed to compare the current density distributions between the untreated and treated conditions. The colored areas represent the spatial extent of the voxels with a significant difference in current density. Log-*F*-ratio statistics were employed, and the color scale represents log-*F*-ratio values (threshold log-*F*-ratio = ±1.414, *p* < 0.05; threshold log-*F*-ratio= ±1.465, *p* < 0.01). A indicates anterior; *P* posterior, *S* superior, *I* inferior, *L* left, *R* right

**Fig. 4:**
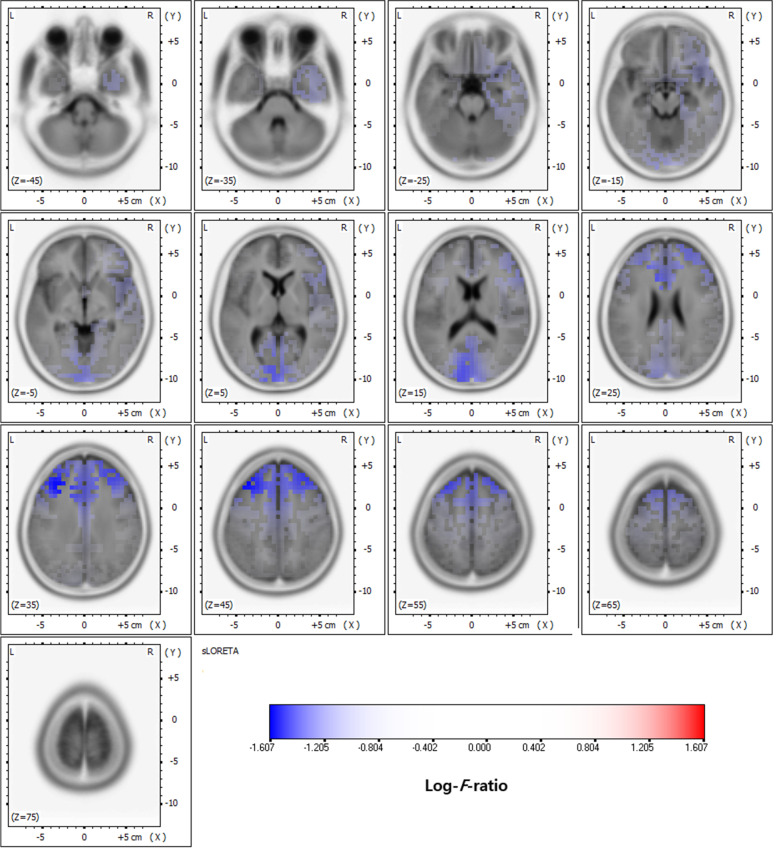
Statistical maps using standardized low-resolution brain electromagnetic tomography (sLORETA) of the theta frequency bands during valproic acid treatment were projected onto a brain magnetic resonance imaging (MRI) template. Nonparametric statistical analysis was performed to compare the current density distributions between the untreated and treated conditions. . The colored areas represent the spatial extent of the voxels with a significant difference in current density. Log-*F*-ratio statistics were employed, and the color scale represents log-*F*-ratio values (threshold log-*F*-ratio = ±1.414, *p* < 0.05; threshold log-*F*-ratio= ±1.465, *p* < 0.01). L indicates left; *R* right

In the delta frequency band, comparative analysis using SnPM of the sLORETA data revealed significant current density differences in the occipital lobe (superior occipital gyrus, middle occipital gyrus, inferior occipital gyrus, cuneus, lingual gyrus, and fusiform gyrus), the parietal lobe (precuneus), and the limbic lobe (posterior cingulate) after six months of VPA treatment. In the theta frequency band, comparative analysis revealed significant current density differences in the frontal lobe (superior frontal gyrus, middle frontal gyrus, medial frontal gyrus, precentral gyrus, and sub-gyral), the occipital lobe (middle occipital gyrus, cuneus, and lingual gyrus), and the limbic lobe (anterior cingulate and posterior cingulate). Otherwise, there was no significant difference. Table 1 lists the regions with significant current density differences (*p* < 0.01 and *p* < 0.05). Figures 1 and 2 respectively show statistical color maps on cortical models and MRI images, respectively, for regions with significant differences in the delta frequency band. Figures 3 and 4 show the corresponding maps in the theta frequency band, respectively.

**Table 1:** Location of significant current density differences in background EEG activity between untreated and treated conditions

Lobe		Hemisphere	*p*-value
*Delta frequency band*			
Occipital	Superior occipital gyrus	left	< 0.01
	Middle occipital gyrus	both	< 0.01
	Inferior occipital gyrus	both	< 0.01 (left), < 0.05 (right)
	Cuneus	both	< 0.01
	Lingual gyrus	both	< 0.01
	Fusiform gyrus	left	< 0.05
Parietal	Precuneus	left	< 0.01
Limbic	Posterior cingulate	left	< 0.01
*Theta frequency band*			
Frontal	Superior frontal gyrus	both	< 0.01
	Middle frontal gyrus	both	< 0.01
	Medial frontal gyrus	both	< 0.01
	Precentral gyrus	both	< 0.01
	Sub-gyral	both	< 0.01
Occipital	Middle Occipital gyrus	both	< 0.01 (left), < 0.05 (right)
	Cuneus	both	< 0.01 (left), < 0.05 (right)
	Lingual gyrus	both	< 0.05
Limbic	Anterior cingulate	both	< 0.01 (left), < 0.05 (right)
	Posterior cingulate	left	< 0.05

The maximum current density difference was found in the cuneus of the left occipital lobe in the delta frequency band (MNI coordinate [x, y, z = −15, −100, 15], Brodmann area 18) (log-*F*-ratio = −1.840, *p* < 0.01) and the medial frontal gyrus of the left frontal lobe in the theta frequency band (MNI coordinate [x, y, z = −35, 30, 45], Brodmann area 9) (log-*F*-ratio = −1.610, *p* < 0.01).

## Discussion

Our results showed that VPA significantly decreased the background EEG activity in low-frequency (delta-theta component) bands across the frontal, parieto-occipital, and limbic lobes.

Several studies have reported background EEG abnormalities and their progress after treatment in epilepsy [31–33]. Some previous studies have also reported background EEG activity changes in patients with JME compared to healthy controls [6, 7, 34]. However, the anatomical extent and frequency bands involved in these studies were inconsistent. Some subsequent studies reported the effects of AED on background EEG activity in patients with JME. Santiago-Rodríguez et al. reported the analysis of background EEG activity in unmedicated and medicated patients with JME [5]. Their results showed various changes according to the anatomical location and frequency of the bands, and these changes were more evident in the frontoparietal region and unmedicated patients. Clemens et al. reported that VPA reduced delta and theta band power in background EEG activity of patients with idiopathic generalized epilepsy [25]. The anatomical distribution was located in the anterior part of the cortex, which includes the entire frontal, anterior temporal, and anterior parietal lobe, insula, and hippocampus.

Recent studies have reported the focal features of JME, predominantly in the frontal lobe [35–37]. Neuropsychological studies also revealed subtle cognitive deficits in patients with JME, indicating frontal lobe dysfunction [10, 38]. In addition, the involvement of other regions, as well as the frontal cortex, has been reported. The sensorimotor and visual cortex areas were particularly involved. These results have been suggested in several functional studies using multimodal methods, such as EEG, MEG, fMRI, and evoked potentials [39–41], consistent with our study. Moreover, our results revealed that AED could reduce the low-frequency band power of the background EEG activity in these regions.

Several explanations can be suggested for our results. Low-frequency background waves on EEG indicate the involvement of functional deficits in the cortical regions [25, 26]. Previous studies reported the functional impairment of JME patients due to the anatomical association of the findings. Our results are a continuation of previous studies and also suggest that antiepileptic treatment would ameliorate these dysfunctions in JME patients. Meanwhile, some studies have reported enhanced low-frequency activity in background EEGs in patients with epilepsy [23, 42, 43]. These changes were interpreted as increased excitability or synchronization that are responsible for seizures and anticonvulsants reduced this activity in the cortical areas involved. JME is a representative generalized epilepsy but generalized seizures do not always develop equally throughout the brain [44]. As noted, the focal features of JME have been reported in several studies. The areas involved were mainly the frontal lobe, and the sensorimotor and visual cortex have often been included [35, 36, 39–41]. We found that the areas involved in those studies closely overlapped with the regions in our results, including the frontal, parieto-occipital, and limbic lobes. Furthermore, limbic lobe involvement was the distinctive feature of our results.

Our study suggested the effect of anticonvulsants on the neural networks involved in JME. To the best of our knowledge, this was the first study using qEEG analysis by the distributed model exclusively in JME. However, our results need to be elucidated in more detail by multimodal techniques, including positron emission tomography, single-photon emission computed tomography, fMRI, and neuropsychological tests. Those multimodal techniques would define the relevant areas in detail, with concepts of the epileptogenic zone, ictal onset zone, symptomatic zone, and irritative zone. In addition, case-control studies, including healthy controls, would further confirm our results.

An additional limitation of this study was the small number of patients. Larger sample size would improve the statistical power and generalizability of the results. However, the SnPM method of sLORETA, which was performed for each contrast with the built-in voxel-wise randomization tests (5000 permutations), was well-suited to our study design and sample size [16, 27]. We used EEG data with 19 electrodes for source localization. The high-density EEG with more electrodes can substantially improve results of source reconstruction. However, only a few clinics are appropriately equipped. In many situations, it is difficult to record EEG with a high number of electrodes. EEG source localization based on recordings with a limited number of electrodes can be better integrated into the clinical setting and broaden the practical applications. sLORETA localization properties have been independently validated [45] and the accuracy has been demonstrated to be similar in high- and low-density EEG [46, 47]. Therefore, we believe that there is a justification for reporting our results.

## Conclusions

In conclusion, this study demonstrated that antiepileptic treatment reduced low-frequency (delta-theta component) background EEG activity in specific areas of patients with JME. In addition, these findings suggest the focal features and the possibility of functional deficits in JME patients. Our results, including the frontal, parieto-occipital, and limbic lobes, were consistent with previous studies and also showed distinctive features. We believe that our results contribute to further understanding the electrophysiological characteristics and AED effects of patients with JME. In the future, more expanded studies are needed including multimodal techniques, age and gender-matched controls, and a larger sample size. Such studies would confirm our results and further expand the understanding of JME.

## Data Availability

The datasets analyzed during the current study are available from the corresponding author on reasonable request.

## Abbreviations

JME: Juvenile myoclonic epilepsy
EEG: Electroencephalography
qEEG: quantitative electroencephalography
VPA: Valproic acid
AED: Antiepileptic drug
MEG: Magnetoencephalography
fMRI: functional magnetic resonance imaging
FFT: Fourier transformation
sLORETA: standardized low-resolution brain electromagnetic tomography
SnPM: Statistical non-parametric mapping
